# Analysis of hemorrhagic transformation and intracerebral hemorrhage under combination therapy with alteplase and antiplatelets or anticoagulants, using the Japanese Adverse Drug Event Report database

**DOI:** 10.1371/journal.pone.0329378

**Published:** 2025-08-18

**Authors:** Tsuyoshi Nakai, Takenao Koseki, Hiroka Nakao, Koki Kato, Kazuo Takahashi, Shigeki Yamada, Shoji Matsumoto

**Affiliations:** 1 Department of Pharmacotherapeutics and Informatics, Fujita Health University School of Medicine, Toyoake, Aichi, Japan; 2 Department of Biomedical Molecular Sciences, Fujita Health University School of Medicine, Toyoake, Aichi, Japan; 3 Department of Comprehensive Strokology, Fujita Health University School of Medicine, Toyoake, Aichi, Japan; Changhua Christian Hospital, TAIWAN

## Abstract

Recombinant tissue-type plasminogen activators (rtPA) effectively dissolve blood clots and improve symptoms in patients with acute ischemic stroke and myocardial infraction. Although rtPA are used in patients taking antiplatelets or anticoagulants to improve clinical outcomes, combination therapy may increase the risk of hemorrhagic transformation (HT) and intracerebral hemorrhage (ICH). However, few studies have investigated the risk of HT and ICH associated with these combination therapies. This study aimed to investigate the adverse-event and drug-drug interaction signals for HT and ICH under combination therapy with alteplase and various antiplatelets or anticoagulants, using the Japanese Adverse Drug Event Report database. Adverse-event signals were evaluated using the reporting odds ratio and information components, and drug-drug interaction signals were studied using the Ω shrinkage measure, additive, multiplicative, and Chi-square statistics models. We also investigated predictors of HT and ICH, time-to-onset, and outcomes in patients receiving alteplase. HT and/or ICH signals were detected in patients receiving alteplase in combination with aspirin, P2Y_12_ inhibitors, cilostazol, ozagrel sodium, direct oral anticoagulants, warfarin potassium, heparin group, or argatroban. Hypertension and diabetes mellitus were significant risk factors for alteplase-induced HT. Most HT and ICH events occurred within 1 day after alteplase administration, and more than 60% of affected patients were not in recovery. In conclusion, continued monitoring is required in patients receiving alteplase in combination with any of the eight types of antiplatelets or the aforementioned anticoagulants. Additionally, the occurrence of HT or ICH within 1 day post-alteplase administration should be considered in patients with hypertension or diabetes mellitus. The findings from this study may help in understanding the risk of HT and ICH induced by rtPA in patients taking antiplatelet or anticoagulant medications, as well as in promoting the appropriate use of rtPA. Further prospective observational studies and randomized controlled trials are needed to assess these finding.

## Introduction

Intravenous recombinant tissue plasminogen activators (rtPA) are used worldwide in patients with acute ischemic stroke (AIS) and myocardial infraction because of its good thrombolytic efficacy [1–3]. rtPA can potently dissolve pathological thrombi in blood vessels by increasing fibrinolytic activity through the conversion of plasminogen to plasmin [4]. However, the injection of rtPA induces various vascular-related adverse events [5,6]. Particularly, the use of rtPA in patients with AIS occasionally increases the risk of intracerebral hemorrhage (ICH) and hemorrhagic transformation (HT), such as symptomatic ICH [5,7]. Antiplatelets, such as aspirin and P2Y_12_ inhibitors, and anticoagulants, such as heparin group and direct oral anticoagulants (DOACs), are often used to prevent thrombus formation and ischemic events in patients with stroke [8,9], coronary artery disease, and atrial fibrillation [10]. rtPA may be administered to patients receiving antiplatelet or anticoagulant therapy for AIS to improve their clinical outcomes. However, the rtPA alteplase has been reported to induce ICH in patients receiving anticoagulants such as DOACs [2,11,12]. Therefore, these combination therapies should be used with caution in patients with AIS. In contrast, there is insufficient evidence regarding the need to limit the combination of alteplase with antiplatelets [2]. Thus, understanding the risks of rtPA-induced HT and ICH in patients on antiplatelet or anticoagulant therapy (especially antiplatelet therapy) is important for promoting the appropriate use of rtPA.

A large pharmacovigilance database is needed to characterize rare adverse drug events. The Japanese Adverse Drug Event Report (JADER) is a large open-access database of spontaneous reports for adverse events (AEs) provided by the Pharmaceuticals and Medical Devices Agency (PMDA) in Japan. The JADER database primarily comprises spontaneous reports submitted by pharmaceutical companies and medical institutions, as well as adverse drug events identified from post-marketing surveillance, clinical trials, and published case reports [13]. The JADER database is useful for detecting AE and drug-drug interaction (DDI) signals in rare cases, such as HT and ICH in patients undergoing treatment with alteplase and antiplatelets or anticoagulants. Several statistical methods for detecting AE and DDI signals have been developed for pharmacovigilance databases and applied to the JADER database [14–16]. Hence, we focused on the JADER database in this study.

Monotherapy with rtPA, including alteplase and tenecteplase, results in AE signals, according to the Food and Drug Administration (FDA) Adverse Event Reporting System (FAERS) database [17,18]. However, it is unclear whether AE signals for HT and ICH occur in patients receiving rtPA in combination with antiplatelets or anticoagulants. The aim of our study was to investigate the AE and DDI signals for HT and ICH under combination therapy with alteplase and various antiplatelets or anticoagulants, using the JADER database. Furthermore, we investigated predictors of HT and ICH, time-to-onset (TTO), and outcomes in patients receiving alteplase.

## Materials and methods

### Data source and study design

**Fig 1:**
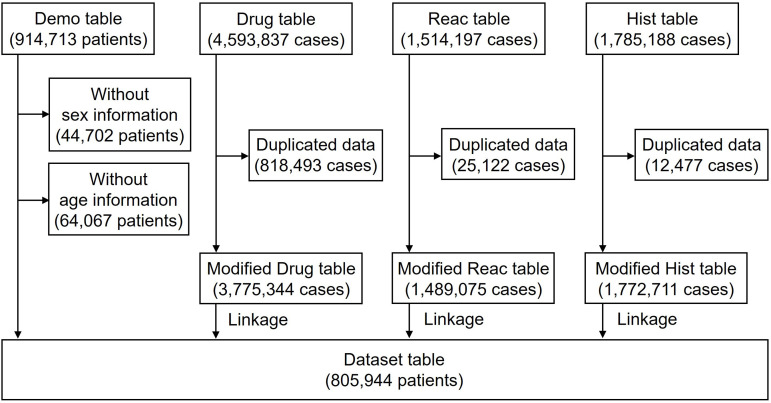
Flow diagram of the study.

Data reported between April 2004 and March 2024 were extracted from the JADER database on the PMDA website [19]. The database comprises four data tables: patient demographic (Demo), drug (Drug), and AE (Reac) information, as well as medical history (Hist). The Drug table was classified into “suspected medication,” “concomitant medicine,” and “interaction,” based on the relevance of the AEs. All categories were used in this study. As age groups in the Demo table were categorized in increments of 10 years, we defined “younger patients” as those in the “under 10s,” “10s,” “20s,” “30s,” “40s,” “50s,” and “60s” age groups and “older patients” as those in the “70s,” “80s,” “90s,” and “100s” age groups [20]. Finally, data from 805,944 patients were analyzed (Fig 1). Two patients treated with alteplase who had the comorbidity of acute myocardial infarction were excluded from the population of patients treated with alteplase, as the focus of all analyses in this study was the use of alteplase in patients with AIS.

Ethics approval and consent to participate were not required because the JADER used for this study is an open-access database.

### Target drugs

The main target drug was alteplase, the only rtPA approved for AIS treatment in Japan. The following 15 antiplatelets used in Japan were included in this study: cyclooxygenase-1 inhibitor, aspirin; P2Y_12_ inhibitors, clopidogrel sulfate, ticagrelor, ticlopidine hydrochloride, and prasugrel hydrochloride; phosphodiesterase-3 inhibitor, cilostazol; phosphodiesterase inhibitor, dipyridamole; thromboxane A2 synthase inhibitor, ozagrel sodium; prostaglandin E_1_ analog, limaprost alfadex; prostaglandin I_2_ analog, beraprost sodium; highly-pure ethyl icosapentate (EPA)-based therapies, EPA and a combination medication of EPA and docosahexaenoic acid, omega-3 fatty acid ethyl; selective 5-hydroxytryptamine 2 receptor antagonist, sarpogrelate hydrochloride; adenosine uptake inhibitor, dilazep hydrochloride hydrate; and platelet-derived growth factor antagonist, trapidil. We selected the following eight anticoagulants used in Japan: DOACs, apixaban, dabigatran etexilate methanesulfonate, edoxaban tosilate hydrate, and rivaroxaban; vitamin K antagonist, warfarin potassium; heparin group, heparin sodium, and heparin calcium; and direct thrombin inhibitor, argatroban.

### Intravenous thrombolysis with alteplase

There are notable differences between the Japanese clinical guidelines and those from the American Heart Association/American Stroke Association (AHA/ASA) regarding the use of intravenous thrombolysis with alteplase in patients. According to the Japanese guidelines, alteplase is administered at a dose of 0.6 mg/kg for AIS within 4.5 h of stroke onset, preferably as early as possible within that time window [12,21,22]. In patients with AIS who are on anticoagulants, alteplase administration is permitted when the prothrombin time-international normalized ratio (PT-INR) is < 1.7 and/or activated partial thromboplastin time (APTT) <1.5 times the baseline value (<40 s as a guide), regardless of the time elapsed since the last anticoagulant dose [12,22]. However, in patients taking DOACs, alteplase administration is not recommended if the last dose was taken within 4 h, even if the coagulation markers meet the above criteria [22].

In contrast, the AHA/ASA guidelines recommend a higher dose of alteplase (0.9 mg/kg) as a Class I, Level A treatment for AIS when administered within 3 h of symptom onset or the time the patient was last observed to be healthy or at baseline, and as early as possible within that window [2]. In patients who have not recently used warfarin, DOACs, or heparin, intravenous thrombolysis can be initiated before obtaining coagulation test results. However, treatment should be discontinued if INR is > 1.7 or PT is abnormally elevated based on local laboratory standards [2]. In patients taking DOACs, alteplase could be considered if relevant coagulation tests, such as APTT, INR, ecarin clotting time, thrombin time, or direct factor Xa activity, are within normal ranges, or if more than 48 h have passed since the last dose and renal function is normal [2].

### Definition of hemorrhagic transformation and intracerebral hemorrhage

HT is generally defined as a hemorrhage at a site of cerebral infarction, following the onset of the latter [23]. ICH is distinguished from HT as a hemorrhage that occurs in a brain region other than a site of cerebral infarction. HT or ICH in the Reac table were coded following preferred terms (PTs) in the Medical Dictionary for Regulatory Activities (MedDRA) version 27.1 J [24]. Cases with the three PTs listed in the S1 Table in S1 File and those with the nine PTs in the S2 Table in S1 File were defined as HT and ICH, respectively, by a stroke physician and a neuroscientist; subsequently, this data was extracted from the Reac table.

### Statistical methods

Multiple methods were employed to achieve optimal signal detection. To evaluate the AE signals of the target drugs for HT or ICH, we calculated the reporting odds ratios (ROR), information components (IC), and 95% confidence intervals (CI) using a two-by-two contingency table (S3 Table in S1 File). The AE signals for HT or ICH were considered positive when the lower limit of the 95% CI of the ROR exceeded 1, and that of the IC exceeded 0. To detect synergistic DDIs, we used the Ω shrinkage measure, additive, multiplicative, and Chi-square statistics models with a four-by-two and a two-by-two contingency table (S4 and S5 Tables in S1 File). The DDI signals for HT or ICH were considered positive when it was detected by at least two of the four DDI models. To evaluate risk factors for HT or ICH with alteplase therapy, multivariable analysis was conducted using logistic regression, considering previous reports [2,23,25–32]. The following covariates were selected: age (older adults [≥70 years old]), sex (male), and comorbidity, including hypertension, diabetes mellitus, heart failure, convulsive disorder, and chronic kidney disease. These comorbidities were extracted from the Hist table based on PTs in the MedDRA version 27.1 J. Each PT was defined according to the narrow scopes of the standardized MedDRA queries (SMQ) for hypertension (code: 20000147), hyperglycaemia/new onset diabetes mellitus (code: 2000041), cardiac failure (code: 20000004), convulsions (code: 20000079), and chronic kidney disease (code: 20000213) (S6–S10 Tables in S1 File). All calculations were performed using Microsoft 365 (Microsoft Corporation, Redmond, WA, United States). Statistical analyses were performed using EZR (Saitama Medical Center, Jichi Medical University, Saitama, Japan), a graphical user interface for R version 4.2.2 (The R Foundation for Statistical Computing, Vienna, Austria) based on a modified version of R Commander (version 1.61) and designed to add statistical functions frequently used in biostatistics [33]. Statistical significance was set at *p *< 0.05.

### Reporting odds ratio

The ROR is an AE signal detection index that is used to compare the odds of reporting a particular AE for a target drug with those of reporting all other AEs for all other drugs [15,17,18,20]. The ROR and 95% CI were calculated using data from the S3 Table in S1 File and Equations (1) and (2). AE signals were considered significant if the lower limit of the 95% CI exceeded 1.


$$ROR\;=\;\frac{N_{11}/N_{10}}{N_{01}/N_{00}}=\;\frac{N_{11}N_{00}}{N_{10}N_{01}}$$ (1)

$$ROR\;(95\%\;CI)\;=e^{\ln(ROR)\;\pm \;1.96\sqrt{\frac{1}{N_{11}}+\;\frac{1}{N_{10}}+\;\frac{1}{N_{01}}+\;\frac{1}{N_{00}}}}$$ (2)

### Information components

The IC of the Bayesian Confidence Propagation Neural Network is an AE signal index based on the Bayesian statistical model. This model can detect AE signals even when the sample size is small [14,15]. The IC and 95% CI were calculated using data from the S3 Table in S1 File and Equations (3–6). AE signals were considered significant when the lower limit of the 95% CI exceeded 0.


$$E\;({IC}_{11})\;=\;\log_{2}\frac{(N_{11}+\;\gamma_{11})(N_{++}\;+\;\alpha )(N_{++}\;+\;\beta )\;}{(N_{++}+\;\gamma )(N_{1+}+\;\alpha_{1})(N_{+1}+\;\beta_{1})\;}$$ (3)

$$V\;\left({IC}_{11}\right)={\left(\frac{1}{ln2}\right)}^{2}[\frac{N_{++}\;-\;N_{11}\;+\;\gamma \;-\;\gamma_{11}}{\left(N_{11}\;+\;\gamma_{11}\right)\left({1\;+\;N}_{++}\;+\;\gamma \right)}+\frac{N_{++}\;-\;N_{1+\;}+\;\alpha \;-\;\alpha_{1}}{\left(N_{1+}\;+\;\alpha_{1}\right)\left({1\;+\;N}_{++}\;+\;\alpha \right)}+\frac{N_{++}\;-\;N_{+1}\;+\;\beta \;-\;\beta_{1}}{\left(N_{+1}\;+\;\beta_{1})({1\;+\;N}_{++}\;+\;\beta \right)};$$ (4)

$$\gamma =\gamma_{11}\frac{\left(N_{++}\;+\;\alpha )(N_{++}\;+\;\beta \right)}{\left(N_{1+}\;+\;\alpha_{1})(N_{+1}\;+\;\beta_{1}\right)},\;\gamma_{11}=1,\;\alpha_{1}=\beta_{1}=1,\;\alpha =\beta =2$$ (5)

$$IC\;(95\%\;CI)=E\left({IC}_{11}\right)\pm 2\sqrt{V\;({IC}_{11})}$$ (6)

### Ω shrinkage measure model

The Ω shrinkage measure is a DDI signal index based on the reporting ratio of a particular AE associated with a two-drug combination to its expected values [16,34,35]. The Ω_025_ values were calculated using data from the S4 Table in S1 File and Equations (7) and (8).


$$\Omega \;=\;log_{2}\frac{n_{111}\;+\;0.5}{E_{111}\;+\;0.5}$$ (7)

$$\Omega_{025}\;=\;\Omega \;-\;\frac{\Phi (0.975)}{\log(2)\sqrt{n_{111}}}$$ (8)

Where E_111_ is the expected value for a particular AE targeted by a two-drug combination, Φ(0.975) is 97.5% of the standard normal distribution, and Ω_025_> 0 is used as a threshold to screen for DDI signals under the combination of two drugs (drug D_1_ and D_2_).

### Additive model

Under the additive assumption, no interaction is established when the excess risk associated with drug D_1_ in the absence of drug D_2_ is same as that associated with drug D_1_ in the presence of drug D_2_ [36]. The signal value of the additive model was calculated using data from the S5 Table in S1 File and Equations (9).


$$p_{11}-p_{00}=(p_{10}+p_{00})+(p_{01}-p_{00})$$ (9)

The signals of potential DDI are detected when $p_{11}-p_{10}-p_{01}+p_{00}>0$

### Multiplicative model

Under the multiplicative model and the assumption of no interaction, the relative risk associated with the concomitant use of drugs D_1_ and D_2_ is equal to the product of the relative risk associated with each individual drug in the absence of the other [36]. The signal value of the multiplicative model was calculated using data from the S5 Table in S1 File and Equation (10).


$$\;\frac{p_{11}}{p_{00}}=\;\frac{p_{10}}{p_{00}}\times \;\frac{p_{01}}{p_{00}}$$ (10)

The signals of potential DDI are detected when $\;\frac{p_{11}\times p_{00}}{p_{10}\times p_{01}}>1$, indicating a positive interaction, where the observed relative risk for the drug combination is significantly greater than the expected risk based on the product of the relative risks for each drug used alone.

### Chi-square statistics model

The Chi-square statistic is a DDI detection model proposed by Gosho et al. [37]. This model is more sensitive to DDI signals than the Ω shrinkage measure method when AEs are rare [37]. The χ was calculated using data from the S4 Table in S1 File and Equation (11).


$$\chi \;=\;\frac{n_{111}\;-\;E_{111}\;-\;0.5}{\sqrt{E_{111}}}$$ (11)

Where χ > 2 is used as the detection criterion to screen for DDI signals under a two-drug combination.

### Analysis of time-to-onset and outcomes of hemorrhagic transformation and intracerebral hemorrhage with alteplase

TTO was calculated using the date of the first alteplase administration in the Drug table and the date of the first occurrence of HT or ICH in the Reac table. We classified cases without the date of the first alteplase administration or that of the first onset of HT or ICH, and those with missing values as unknown. Additionally, the analysis period for TTO of alteplase-induced HT or ICH was 20 days; cases in which these AEs occurred after this period were excluded. TTO analysis was performed using JMP 13.0 (SAS Institute Inc., Cary, NC, USA). The outcomes were examined using the Reac table. Cases with missing information were classified as unknown.

## Results

### Hemorrhagic transformation and intracerebral hemorrhage signals under concomitant therapy

S11 and S12 Tables in S1 File present the RORs and ICs for HT and ICH, respectively, under monotherapy with alteplase, antiplatelets, and anticoagulants. HT and ICH signals were detected for alteplase (HT: ROR, 645.83 [95% CI 554.19752.63]; IC, 7.26 [95% CI 7.05 to 7.46]; ICH: ROR, 29.91 [95% CI 25.72 to 34.77]; IC, 4.33 [95% CI 4.12 to 4.54]).

**Fig 2:**
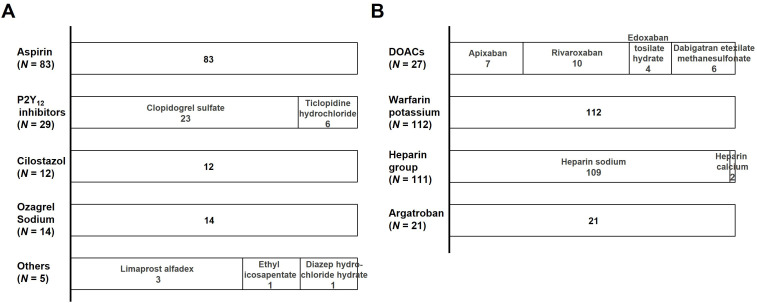
The distribution of the pharmacological class of antiplatelets (A) and anticoagulants (B). N, number of cases; Others, other types of antiplatelets; DOACs, direct oral anticoagulants.

Among patients who received alteplase in combination with antiplatelets or anticoagulants, there was at least one reported AEs for alteplase when administered in combination with the following eight antiplatelets: aspirin, clopidogrel sulfate, ticlopidine hydrochloride, cilostazol, ozagrel sodium, limaprost alfadex, ethyl icosapentate, and dilazep hydrochloride hydrate; as well as the following all eligible anticoagulants: apixaban, rivaroxaban, edoxaban tosilate hydrate, dabigatran etexilate methanesulfonate, warfarin potassium, heparin sodium, heparin calcium, and argatroban (Fig 2). To reduce the risk of false-positive or negative AE signals caused by the limited number of individual case reports, these drugs were categorized into the following five groups of antiplatelets: aspirin, P2Y_12_ inhibitors, cilostazol, ozagrel sodium, and other types of antiplatelets (others); and the following four groups of anticoagulants: DOACs, warfarin potassium, heparin group, and argatroban (Fig 2).

The RORs and ICs for HT and ICH in patients who received alteplase in combination with each group of antiplatelets or anticoagulants are summarized in Table 1. Alteplase showed HT signals when administered in combination with the following four types of antiplatelets: aspirin (ROR, 413.60 [95% CI 262.33 to 652.10]; IC, 4.75 [95% CI 4.13 to 5.37]), P2Y_12_ inhibitors (ROR, 398.13 [95% CI 184.69 to 858.22]; IC, 3.40 [95% CI 2.38 to 4.42]), cilostazol (ROR, 376.10 [95% CI 113.08 to 1250.89]; IC, 2.30 [95% CI 0.78 to 3.82]), and ozagrel sodium (ROR, 1006.71 [95% CI 348.70 to 2906.39]; IC, 3.14 [95% CI 1.92 to 4.36]). HT signals were shown when combined with the following all types of anticoagulants: DOACs (ROR, 171.11 [95% CI 64.68 to 452.70]; IC, 2.53 [95% CI 1.23 to 3.83]), warfarin potassium (ROR, 525.31 [95% CI 358.27 to 770.21]; IC, 5.32 [95% CI 4.81 to 5.83]), heparin group (ROR, 421.19 [95% CI 283.91 to 624.85]; IC, 5.12 [95% CI 4.58 to 5.66]), and argatroban (ROR, 566.80 [95% CI 238.33 to 1348.00]; IC, 3.28 [95% CI 2.18 to 4.38]).

**Table 1:** Reporting odds ratio and information components for hemorrhagic transformation (HT) and intracerebral hemorrhage (ICH) in patients receiving alteplase treatment in combination with antiplatelets or anticoagulants.

		Total AEs	Cases	ROR [95% CI]	IC [95% CI]	Signal
**HT**						
Alteplase	Aspirin	83	29	413.60 [262.33 to 652.10]	4.75 [4.13 to 5.37]	Yes
	P2Y_12_ inhibitors	29	10	398.13 [184.69 to 858.22]	3.40 [2.38 to 4.42]	Yes
	Cilostazol	12	4	376.10 [113.08 to 1250.89]	2.30 [0.78 to 3.82]	Yes
	Ozagrel sodium	14	8	1006.71 [348.70 to 2906.39]	3.14 [1.92 to 4.36]	Yes
	Others	5	1	187.53 [20.94 to 1679.23]	0.99 [−1.37 to 3.35]	No
	DOACs	27	5	171.11 [64.68 to 452.70]	2.53 [1.23 to 3.83]	Yes
	Warfarin potassium	112	45	525.31 [358.27 to 770.21]	5.32 [4.81 to 5.83]	Yes
	Heparin group	111	39	421.19 [283.91 to 624.85]	5.12 [4.58 to 5.66]	Yes
	Argatroban	21	9	566.80 [238.33 to 1348.00]	3.28 [2.18 to 4.38]	Yes
**ICH**						
Alteplase	Aspirin	83	27	40.83 [25.78 to 64.65]	3.82 [3.19 to 4.45]	Yes
	P2Y_12_ inhibitors	29	13	68.70 [33.04 to 142.86]	3.37 [2.44 to 4.31]	Yes
	Cilostazol	12	4	42.24 [12.72 to 140.29]	2.12 [0.60 to 3.64]	Yes
	Ozagrel sodium	14	2	14.08 [3.15 to 62.90]	1.35 [−0.47 to 3.18]	No
	Others	5	1	21.11 [2.36 to 188.91]	0.90 [−1.45 to 3.26]	No
	DOACs	27	8	35.58 [15.57 to 81.30]	2.76 [1.65 to 3.87]	Yes
	Warfarin potassium	112	19	17.28 [10.55 to 28.32]	3.11 [2.41 to 3.81]	Yes
	Heparin group	111	22	20.92 [13.11 to 33.37]	3.32 [2.65 to 3.98]	Yes
	Argatroban	21	4	19.88 [6.69 to 59.08]	1.99 [0.56 to 3.42]	Yes

AEs, adverse events; ROR, reporting odds ratio; IC, information component; CI, confidence interval; Others, other types of antiplatelets; DOACs, direct oral anticoagulants.

In addition, ICH signals were observed when alteplase was combined with the following three types of antiplatelets: aspirin (ROR, 40.83 [95% CI 25.78 to 64.65]; IC, 3.82 [95% CI 3.19 to 4.45]), P2Y_12_ inhibitors (ROR, 68.70 [95% CI 33.04 to 142.86]; IC, 3.37 [95% CI 2.44 to 4.31]), and cilostazol (ROR, 42.24 [95% CI 12.72 to 140.29]; IC, 2.12 [95% CI 0.60 to 3.64]). ICH signals were also observed when combined with the following all types of anticoagulants: DOACs (ROR, 35.58 [95% CI 15.57 to 81.30]; IC, 2.76 [95% CI 1.65 to 3.87]), warfarin potassium (ROR, 17.28 [95% CI 10.55 to 28.32]; IC, 3.11 [95% CI 2.41 to 3.81]), heparin group (ROR, 20.92 [95% CI 13.11 to 33.37]; IC, 3.32 [95% CI 2.65 to 3.98]), and argatroban (ROR, 19.88 [95% CI 6.69 to 59.08]; IC, 1.99 [95% CI 0.56 to 3.42]).

### Drug-drug interactions for hemorrhagic transformation and intracerebral hemorrhage between alteplase and antiplatelets or anticoagulants

The DDI signals for HT and ICH are summarized in Table 2. No DDI signals for HT and ICH were detected between alteplase and any type of antiplatelets or anticoagulants. However, in the additive model only, warfarin potassium (the signal value based on the additive model [AM]: 0.01) showed an interactive signal for HT when used in combination with alteplase. In the same model, P2Y_12_ inhibitors (AM: 0.01), cilostazol (AM: 0.02), and others (AM: 0.02) showed interactive signals for ICH in combination with alteplase. No interactive signals were detected for any type of antiplatelets or anticoagulants in the multiplicative, the Ω shrinkage measure, and the Chi-square statistics models.

**Table 2:** The drug-drug interaction signals of alteplase with antiplatelets or anticoagulants for HT and ICH.

		*n*_11+_	*n*_111_	*E*_111_	Ω_025_	AM	MM	χ	Signal
**HT**									
Alteplase	Aspirin	219	72	79.17	−0.47	−0.03	0.16	−0.86	No
	P2Y_12_ inhibitors	112	37	40.23	−0.58	−0.03	0.18	−0.59	No
	Cilostazol	55	10	19.99	−1.86	−0.18	0.10	−2.35	No
	Ozagrel sodium	40	14	14.53	−0.81	−0.02	0.03	−0.27	No
	Others	15	3	5.37	−2.38	−0.16	0.31	−1.23	No
	DOACs	46	10	16.70	−1.61	−0.15	0.03	−1.76	No
	Warfarin potassium	285	104	101.24	−0.24	0.01	0.27	0.22	No
	Heparin group	305	92	113.31	−0.59	−0.07	0.06	−2.05	No
	Argatroban	67	19	25.57	−1.07	−0.13	0.01	−1.40	No
**ICH**									
Alteplase	Aspirin	219	53	55.24	−0.45	−0.02	0.30	−0.37	No
	P2Y_12_ inhibitors	112	30	28.37	−0.44	0.01	0.30	0.21	No
	Cilostazol	55	16	13.99	−0.52	0.02	0.33	0.40	No
	Ozagrel sodium	40	8	11.23	−1.47	−0.11	0.12	−1.11	No
	Others	15	4	3.63	−1.29	0.02	0.68	−0.07	No
	DOACs	46	13	14.35	−0.92	−0.08	0.06	−0.49	No
	Warfarin potassium	285	49	77.26	−1.06	−0.11	0.16	−3.27	No
	Heparin group	305	53	81.05	−1.00	−0.10	0.22	−3.17	No
	Argatroban	67	8	18.42	−2.15	−0.18	0.08	−2.54	No

HT, hemorrhagic transformation; ICH, intracerebral hemorrhage; *n*_11+_, the reported number of total adverse events induced by two-drug combinations; *n*_111_, the reported number of adverse events targeted by two-drug combinations; *E*_111_, the expected value of adverse events targeted by two-drug combinations; Ω_025_, the signal value based on the Ω shrinkage measure model; AM, the signal value based on the additive model; MM, the signal value based on the multiplicative model; χ, the signal value based on the Chi-square statistics model; Others, other types of antiplatelets; DOACs, direct oral anticoagulants.

### Predictors for hemorrhagic transformation and intracerebral hemorrhage with alteplase treatment

Multivariable logistic regression analysis was performed to investigate the predictors of alteplase-induced HT or ICH (Table 3). The analysis revealed that hypertension and diabetes mellitus were independent predictors for alteplase-induced HT (hypertension: OR, 1.65 [95% CI = 1.24 to 2.20], *p* < 0.001; diabetes mellitus: OR, 1.75 [95% CI 1.20 to 2.55], *p* = 0.004). Significant predictors of alteplase-induced ICH were not identified in multivariable analysis; however, the convulsive disorder was not included as a variable in the analysis because this comorbidity was not observed in any patients with alteplase-induced ICH. Additionally, multivariable logistic regression analysis was not applied to cases of concomitant therapy with alteplase in combination with antiplatelets or anticoagulants owing to a limited sample size.

**Table 3:** Multivariable logistic analysis of predictors for alteplase induced-HT or alteplase induced-ICH.

	Multivariable analysis	
	OR [95% CI]	*p*-Value
HT		
Male	1.27 [0.95 to 1.70]	0.102
Older adults	0.99 [0.74 to 1.33]	0.942
Hypertension	1.65 [1.24 to 2.20]	< 0.001
Diabetes mellitus	1.75 [1.20 to 2.55]	0.004
Heart failure	1.44 [0.78 to 2.66]	0.244
Convulsive disorder	0.75 [0.07 to 8.46]	0.819
Chronic kidney disease	0.60 [0.18 to 2.02]	0.409
ICH		
Male	1.07 [0.85 to 1.35]	0.569
Older adults	1.14 [0.90 to 1.45]	0.271
Hypertension	0.90 [0.66 to 1.24]	0.537
Diabetes mellitus	0.67 [0.42 to 1.06]	0.089
Heart failure	0.62 [0.28 to 1.36]	0.230
Convulsive disorder	N.A.	
Chronic kidney disease	0.26 [0.03 to 1.99]	0.193

HT, hemorrhagic transformation; ICH, intracerebral hemorrhage; OR, odds ratio; CI, confidence intervals; N.A., not applicable.

### Time-to-onset and outcomes of hemorrhagic transformation and intracerebral hemorrhage with alteplase

**Fig 3:**
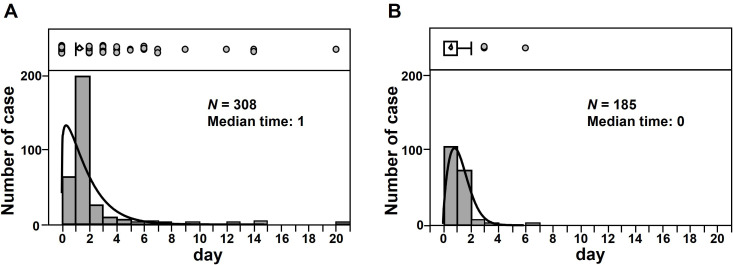
Time-to-onset for alteplase-induced hemorrhagic transformation (A) and intracerebral hemorrhage (B). N, number of cases.

Data from 308 of 336 patients with alteplase-induced HT were used for evaluating TTO (Fig 3A and Table 4). Among the 308 patients, 197 (63%) experienced a TTO of 1 day post-alteplase administration. The median time for alteplase-induced HT was 1 day. The outcomes were analyzed in 307 patients with alteplase-induced HT (Table 4), among whom 94 (31%) were in recovery or remission and 213 (69%) were not in recovery. Among 185 patients with alteplase-induced ICH, 103 (56%) exhibited a TTO of 0 days (the day of alteplase administration) (Fig 3B and Table 4). The median time for alteplase-induced ICH was 0 days. Additionally, the outcomes were analyzed for 187 patients with alteplase-induced ICH (Table 4); among them, 31 (17%) were in recovery or remission and 156 (83%) were not.

**Table 4:** Time-to-onset and outcomes of HT and ICH in patients with alteplase.

Time-to-onset (day)	*N*	Outcomes	*N*
HT			
0	62	Recovery	24
1	197	Remission	70
2	24	No recovery	69
3	8	After-effect	59
4-20	17	Death	85
Unknown	28	Unknown	29
ICH			
0	103	Recovery	11
1	72	Remission	20
2	6	No recovery	28
3	2	After-effect	74
4-20	2	Death	54
Unknown	45	Unknown	45

Time-to-onset was defined as the period from the first alteplase administration to the date of the first occurrence of HT or ICH. HT, hemorrhagic transformation; ICH, intracerebral hemorrhage; N, number of cases.

## Discussion

The main findings of the present study were as follows: (1) HT and ICH signals were detected for alteplase administered in combination with several types of antiplatelets and anticoagulants, whereas DDI signals for HT and ICH were not. (2) Hypertension and diabetes mellitus were independent predictive factors for alteplase-induced HT. (3) The results of the TTO analyses for alteplase-induced HT and alteplase-induced ICH were different.

Consistent with previous findings [5,7], HT and ICH signals were detected following alteplase monotherapy (S11 and S12 Tables in S1 File). Furthermore, we demonstrated that alteplase showed HT and/or ICH signals when administered in combination with the antiplatelets aspirin, P2Y_12_ inhibitors, cilostazol, or ozagrel sodium (Table 1). Pretreatment with several antiplatelets has been reported to increase the risk of HT and ICH in patients receiving alteplase [26,38,39]. A growing number of studies report that alteplase therapy increases the risk of HT and ICH, especially in patients receiving aspirin [26–29,40,41]. A retrospective analysis showed that clopidogrel sulfate treatment significantly increased the risk of HT in patients treated with alteplase, compared with the absence of antiplatelet treatment [42]. Another analysis showed that patients receiving antiplatelet agents other than aspirin, mainly ticlopidine hydrochloride, were at high risk of alteplase-induced HT [26], although the safety of combination therapy with alteplase and P2Y_12_ inhibitors other than clopidogrel sulfate has not been well reported. These findings were consistent with our results. However, several studies have shown contradictory results that alteplase, in combination with clopidogrel sulfate, was safe in clinical practice [29,40,41]. Therefore, further prospective observational studies and randomized controlled trials reporting the combination therapy of alteplase and P2Y_12_ inhibitors are needed to assess the results of this study. HT and/or ICH signals were detected in patients receiving alteplase in combination with cilostazol or ozagrel sodium, despite a lack of evidence regarding the risk of HT and ICH with these combination therapies. Thus, the risk of HT and ICH should be considered in patients receiving alteplase in combination with cilostazol or ozagrel sodium. Neither HT nor ICH signals were detected in patients receiving alteplase in combination with other types of antiplatelets (others) other than aspirin, P2Y_12_ inhibitors, ozagrel sodium, and cilostazol. For all AEs reported in the JADER, only a small number of cases were combination therapy with alteplase and other types of antiplatelets; therefore, the present results for these combination therapies may need to be reevaluated using a larger medical or pharmacovigilance database.

Current Japanese guidelines permit administering alteplase to patients with AIS taking anticoagulants with a PT-INR < 1.7 and/or APTT <1.5 times the baseline value [12,22]. In patients taking DOACs, alteplase administration is not recommended if the last dose was taken within 4 h, even if the coagulation markers meet the above criteria [22]. In the present study, HT and ICH signals were detected in patients on alteplase in combination with the anticoagulants DOACs, warfarin potassium, heparin group, and argatroban (Table 1). Alteplase in combination with DOACs, warfarin potassium, heparin group, or argatroban have been shown to increase the risk of HT and/or ICH [11,12,43–48]. Our findings support these results of previous clinical or epidemiological studies.

In contrast, these combinations are reportedly safe when used following the guidelines for alteplase use [49–56]. However, in the present study, it was unclear whether these combination therapies were administered to all patients according to the guideline because the JADER database does not include detailed clinical information. In many cases of each combination therapy, the date of initial administration or the drug dosage were missing. Protocol violations with the use of alteplase may increase the risk of HT and ICH [57]. These limitations show that the risk of HT and ICH induced by alteplase monotherapy or combination therapy with antiplatelets or anticoagulants may be overestimated in this study, which may limit the applicability of our findings to clinical practice. Therefore, our findings should be interpreted with caution. The risk of HT and ICH associated with these combination therapies requires further investigation.

Our findings reveal that the risk of HT and ICH may increase when alteplase is used in combination with antiplatelets or anticoagulants; nevertheless, these results should be interpreted in the context of the well-established clinical benefits of alteplase in patients with AIS. According to AHA/ASA guidelines [2], intravenous alteplase is recommended as a Class I, Level A treatment for AIS, ideally administered as early as possible and within 3 h of symptom onset or the last known time of wellness, based on strong evidence of improved outcomes [6,58–64]. The overall benefit-risk profile of alteplase remains favorable, particularly when administered to patients who are appropriately selected and guidelines are followed. Our findings highlight the need for careful monitoring and individualized risk assessment; however, they do not diminish the established clinical value of alteplase in eligible patients.

Analysis of the safety profile of DDIs is challenging but important because the proportion of AEs attributed to DDIs is estimated to be approximately 30% of unexpected AEs [65]. In this study, the Ω shrinkage measure, additive, multiplicative, and Chi-square statistics models were applied to alteplase and all types of antiplatelets and anticoagulants to detect synergistic DDIs that could lead to HT or ICH (Table 2). Against our expectations, no drug combination showed synergistic interactions for HT or ICH development, despite the detection of HT and ICH signals with the respective monotherapies (S11 and S12 Tables in S1 File). This result may be partly attributed to the small number of cases for each drug combination. Moreover, for the same reason, further subgroup analysis for DDI detection was difficult. In addition to the limited number of cases, this result may also reflect the inherent limitations of current DDI models. Noguchi *et al* reported that the Ω shrinkage measure model is the most conservative and tends to detect the fewest DDI signals among the four models used in this study [16]. In contrast, the additive and multiplicative models are more likely to detect positive signals even when the number of cases is small. The Chi-square statistics model is considered more sensitive to rare events; meanwhile, its detection pattern was very similar to that of the Ω shrinkage measure model. The characteristics of the four DDI models should be considered when interpreting the lack of DDI signals in our results. In this study, weak interactive signals for HCI or ICH were detected using the additive model when alteplase was used in combination with warfarin potassium, P2Y_12_ inhibitors, cilostazol, or other types of antiplatelets. However, these signals were not confirmed by the Ω shrinkage measure model. Therefore, our results are insufficient for concluding the absence of synergistic interactions for the development of HT or ICH with these combination therapies. In the future, the results of this study should be reevaluated in a larger population using more comprehensive medical or pharmacovigilance databases.

Alteplase-induced HT was significantly associated with hypertension and diabetes mellitus in our analysis (Table 3). These findings are supported by those from previous studies, which have shown that hypertension, elevated systolic blood pressure, diabetes mellitus, and hyperglycemia are each independently associated with an increased risk of alteplase-induced HT [23,26–28]. These vascular and metabolic conditions may compromise the integrity of the blood-brain barrier or exacerbate reperfusion injury, thereby increasing the likelihood of bleeding complications [66]. The AHA/ASA guidelines also emphasize the importance of strict control of blood pressure and glucose levels before administering alteplase [2]. Our findings are consistent with these reports and guidelines, supporting the need for careful management of blood pressure and glucose levels before initiating treatment.

The median time of occurrence of HT and ICH was 1 and 0 days after alteplase administration, respectively, and most of these events occurred within 1 day (Fig 3 and Table 4). Lopez-Yunez et al. demonstrated that most HT and ICH events occurred within 36 h after alteplase administration, consistent with our findings [67]. Additionally, we observed that the mode values for HT and ICH were 1 and 0 days after alteplase administration, respectively. These results may reflect the differences in the development patterns of HT and ICH. Moreover, more than 60% of the patients who developed alteplase-induced HT and ICH were not in recovery (Table 4), suggesting poor prognosis following the development of these AEs. Based on our results and previous reports, alteplase should be administered with caution, followed by continuous monitoring.

Our study has certain limitations. First, spontaneous reporting systems, such as JADER, have various biases, including under- or over-reporting, the Weber effect [68], the ripple effect, notoriety bias [69], and the masking effect [70], as well as confounding by comorbidities. Additionally, the JADER database tends to be biased toward the reporting of serious AEs [15]. As these biases could not be ruled out in this study, they may affect the reliability of the data obtained from this large spontaneous reporting system. Consequently, the JADER database may reflect only a limited portion of the AEs that occur in real-world clinical practice. Therefore, signals, including inverse signals, should be interpreted with caution [71]. Second, clinical data at the time of alteplase administration were inadequate. For example, neurological signs, medical conditions, blood findings, and adherence of guidelines at the time of alteplase administration were not registered in the JADER database. Furthermore, many cases for each combination therapy had missing values for the date of first administration or drug dose. These factors are considered important in the development of alteplase-induced HT or ICH; nevertheless, we were unable to evaluate them owing to the lack of data. Third, disproportionality analysis was difficult for combination therapy with three drugs, such as alteplase and dual antiplatelet agents (e.g., aspirin and clopidogrel sulfate), because, to the best of our knowledge, no appropriate model exists for evaluating the interactions among more than three drugs. Finally, because of the lack of distinction between past medical history and primary diseases in the JADER database, we were unable to analyze whether patients treated with alteplase had a history of cerebrovascular disorders. Further prospective observational studies and randomized controlled trials are needed to access our results.

## Conclusions

HT and/or ICH signals were detected in patients receiving alteplase in combination with aspirin, P2Y_12_ inhibitors, cilostazol, ozagrel sodium, DOACs, warfarin potassium, heparin group, or argatroban. Therefore, continued monitoring is required, particularly for these combination therapies. Additionally, the occurrence of HT or ICH within 1 day post-alteplase administration should be carefully considered, especially in patients treated with alteplase who have hypertension or diabetes mellitus. However, these findings do not undermine the established clinical value of alteplase when administered in accordance with clinical guidelines. Our results may help in understanding the risk of HT and ICH induced by the use of rtPA in patients taking antiplatelet or anticoagulant medications, as well as in promoting the appropriate use of rtPA. Future prospective observational studies and randomized controlled trials are necessary to assess these findings.

## Supporting information

S1 File S1 Table. Definition of hemorrhagic transformation (HT). S2 Table. Definition of intracerebral hemorrhage (ICH). S3 Table. Two-by-two contingency table for adverse-event signal detection. S4 Table. Four-by-two contingency table for drug-drug interaction signal detection. S5 Table. Two-by-two contingency table for drug-drug interaction signal detection. S6 Table. Definition of hypertension. S7 Table. Definition of diabetes mellitus. S8 Table. Definition of heart failure. S9 Table. Definition of convulsions. S10 Table. Definition of chronic kidney disease. S11 Table. Reporting odds ratio and information components of HT for each drug as monotherapy. S12 Table. Reporting odds ratio and information components of ICH for each drug as monotherapy.
(ZIP)
