# Supplementary material for: Analysis of hemorrhagic transformation and intracerebral hemorrhage under combination therapy with alteplase and antiplatelets or anticoagulants, using the Japanese Adverse Drug Event Report database
Source: PLoS One. 2025 Aug 18;20(8):e0329378. doi: 10.1371/journal.pone.0329378 (PMC12360569; doi:10.1371/journal.pone.0329378)
Supplement: S1 File — S1 Table. Definition of hemorrhagic transformation (HT). S2 Table. Definition of intracerebral hemorrhage (ICH). S3 Table. Two-by-two contingency table for adverse-event signal detection. S4 Table. Four-by-two contingency table for drug-drug interaction signal detection. S5 Table. Two-by-two contingency table for drug-drug interaction signal detection. S6 Table. Definition of hypertension. S7 Table. Definition of diabetes mellitus. S8 Table. Definition of heart failure. S9 Table. Definition of convulsions. S10 Table. Definition of chronic kidney disease. S11 Table. Reporting odds ratio and information components of HT for each drug as monotherapy. S12 Table. Reporting odds ratio and information components of ICH for each drug as monotherapy. (ZIP) [file pone.0329378.s001.zip › Supporting Information file/S10 Table.pdf]

S10 Table. Definition of chronic kidney disease.

SMQ code	SMQ name
20000213	Chronic kidney disease
PT code	PT name
10087686	APOL1-mediated kidney disease
10053699	Artificial kidney device user
10003885	Azotaemia
10064848	Chronic kidney disease
10091062	Chronic kidney disease-associated pruritus
10078095	Chronic kidney disease-mineral and bone disorder
10010082	Coma uraemic
10012660	Diabetic end stage renal disease
10061105	Dialysis
10059015	Dialysis device insertion
10077512	End stage renal disease
10083258	Erythropoietin deficiency anaemia
10018367	Glomerulonephritis chronic
10018875	Haemodialysis
10053090	Haemofiltration
10019845	Hepatorenal failure
10062624	High turnover osteopathy
10020708	Hyperparathyroidism secondary
10023421	Kidney fibrosis
10063000	Low turnover osteopathy
10081588	Metabolic nephropathy
10058116	Nephrogenic anaemia
10067467	Nephrogenic systemic fibrosis
10029159	Nephrosclerosis
10049630	Oedema due to renal disease
10034498	Pericarditis uraemic
10034660	Peritoneal dialysis
10052279	Renal and liver transplant
10052278	Renal and pancreas transplant
10087816	Renal artery revascularization
10038435	Renal failure

S10 Table (continued).

10074746	Renal replacement therapy
10038519	Renal rickets
10038533	Renal transplant
10056609	Uraemia odour
10046324	Uraemic acidosis
10087409	Uraemic cardiomyopathy
10046326	Uraemic encephalopathy
10063709	Uraemic gastropathy
10077910	Uraemic myopathy
10046328	Uraemic neuropathy
10067863	Uridrosis

SMQ, standardized Medical Dictionary for Regulatory Activities (MedDRA) queries; PT, preferred term.
