# Supplementary material for: Analysis of hemorrhagic transformation and intracerebral hemorrhage under combination therapy with alteplase and antiplatelets or anticoagulants, using the Japanese Adverse Drug Event Report database
Source: PLoS One. 2025 Aug 18;20(8):e0329378. doi: 10.1371/journal.pone.0329378 (PMC12360569; doi:10.1371/journal.pone.0329378)
Supplement: S1 File — S1 Table. Definition of hemorrhagic transformation (HT). S2 Table. Definition of intracerebral hemorrhage (ICH). S3 Table. Two-by-two contingency table for adverse-event signal detection. S4 Table. Four-by-two contingency table for drug-drug interaction signal detection. S5 Table. Two-by-two contingency table for drug-drug interaction signal detection. S6 Table. Definition of hypertension. S7 Table. Definition of diabetes mellitus. S8 Table. Definition of heart failure. S9 Table. Definition of convulsions. S10 Table. Definition of chronic kidney disease. S11 Table. Reporting odds ratio and information components of HT for each drug as monotherapy. S12 Table. Reporting odds ratio and information components of ICH for each drug as monotherapy. (ZIP) [file pone.0329378.s001.zip › Supporting Information file/S11 Table.pdf]

S11 Table. Reporting odds ratio and information components of HT for each drug as monotherapy.

Pharmacological classification	Drugs	N_{11}	N_{10}	N_{01}	N_{00}	ROR [95% CI]	IC [95% CI]	Signal
**Intravenous thrombolysis**								
tPA	Alteplase	336	567	738	804,303	645.832 [554.19 to 752.63]	7.26 [7.05 to 7.46]	Yes
**Antiplatelets**								
COX-1 inhibitor	Aspirin	24	20,101	1,050	784,769	0.89 [0.60 to 1.34]	-0.16 [-0.74 to 0.43]	No
$P2Y_{12}$ inhibitor	Clopidogrel sulfate	10	5,683	1,064	799,187	1.32 [0.71 to 2.45]	0.36 [-0.52 to 1.23]	No
	Ticlopidine hydrochloride	1	1,555	1,073	803,315	0.48 [0.07 to 3.42]	-0.62 [-2.66 to 1.42]	No
	Prasugrel hydrochloride	0	168	1,074	804,702	0	-0.29 [-3.19 to 2.60]	No
	Ticagrelor	0	4	1,074	804,866	0	-0.01 [-3.17 to 3.15]	No
PDE-3 inhibitor	Cilostazol	4	3,475	1,070	801,395	0.86 [0.32 to 2.30]	-0.17 [-1.47 to 1.12]	No
PDE inhibitor	Dipyridamole	0	1,134	1,074	803,736	0	-1.33 [-4.22 to 1.56]	No
TXA2 synthase inhibitor	Ozagrel sodium	3	261	1,071	804,609	8.64 [2.76 to 26.99]	1.56 [0.11 to 3.02]	Yes
PGE_1 analogue	Limaprost alfadex	1	3,077	1,073	801,793	0.24 [0.03 to 1.73]	-1.35 [-3.40 to 0.69]	No
PGI_2 analogue	Beraprost sodium	0	1,060	1,074	803,810	0	-1.27 [-4.16 to 1.62]	No
EPA	Ethyl icosapentate	0	2,391	1,074	802,479	0	-2.07 [-4.95 to 0.82]	No
EPA and DHA	Omega-3 fatty acid ethyl	1	601	1,073	804,269	1.25 [0.18 to 8.88]	0.15 [-1.90 to 2.19]	No
$5\text{-}HT_2$ receptor antagonist	Sarpogrelate hydrochloride	0	671	1,074	804,199	0	-0.92 [-3.81 to 1.97]	No
Adenosine uptake inhibitor	Dilazep hydrochloride hydrate	0	371	1,074	804,499	0	-0.58 [N.A. to N.A.]	No
PDGF antagonist	Trapidil	0	98	1,074	804,772	0	-0.17 [-3.08 to 2.72]	No
**Anticoagulants**								
DOACs	Apixaban	49	8,148	1,025	796,722	4.67 [3.51 to 6.23]	2.07 [1.65 to 2.49]	Yes
	Rivaroxaban	52	5,903	1,022	798,967	6.89 [5.21 to 9.11]	2.57 [2.16 to 2.97]	Yes
	Edoxaban tosilate hydrate	16	4,934	1,058	799,936	2.45 [1.50 to 4.02]	1.16 [0.45 to 1.87]	Yes
	Dabigatran etexilate methanesulfonate	23	3,060	1,051	801,810	5.73 [3.79 to 8.68]	2.23 [1.63 to 2.83]	Yes
Vitamin K antagonist	Warfarin potassium	11	10,582	1,063	794,288	0.78 [0.43 to 1.41]	-0.33 [-1.17 to 0.50]	No
Heparin group	Heparin sodium	26	5,301	1,048	799,569	3.74 [2.53 to 5.53]	1.74 [1.17 to 2.30]	Yes
	Heparin calcium	4	707	1,070	804,163	4.25 [1.59 to 11.38]	1.36 [0.06 to 2.66]	Yes
Direct thrombin inhibitor	Argatroban	10	182	1,064	804,688	41.55 [22.92 to 78.76]	3.13 [2.23 to 4.03]	Yes

S11 Table (continued).

N_{11}, N_{10}, N_{01}, and N_{00} are shown in S3 Table, respectively. HT, hemorrhagic transformation; ROR, reporting odds ratio; IC, information component; CI, confidence interval; tPA, tissue-type plasminogen activator, COX-1, cyclooxygenase-1; N.A., not applicable; PDE-3, phosphodiesterase-3; TXA2, thromboxane A2; PGE_1, prostaglandin E_1; EPA, ethyl icosapentate; DHA, docosahexaenoic acid; 5-HT_2, 5-hydroxytryptamine 2; PDGF, platelet-derived growth factor; DOACs, direct oral anticoagulants.
