# Supplementary material for: Analysis of hemorrhagic transformation and intracerebral hemorrhage under combination therapy with alteplase and antiplatelets or anticoagulants, using the Japanese Adverse Drug Event Report database
Source: PLoS One. 2025 Aug 18;20(8):e0329378. doi: 10.1371/journal.pone.0329378 (PMC12360569; doi:10.1371/journal.pone.0329378)
Supplement: S1 File — S1 Table. Definition of hemorrhagic transformation (HT). S2 Table. Definition of intracerebral hemorrhage (ICH). S3 Table. Two-by-two contingency table for adverse-event signal detection. S4 Table. Four-by-two contingency table for drug-drug interaction signal detection. S5 Table. Two-by-two contingency table for drug-drug interaction signal detection. S6 Table. Definition of hypertension. S7 Table. Definition of diabetes mellitus. S8 Table. Definition of heart failure. S9 Table. Definition of convulsions. S10 Table. Definition of chronic kidney disease. S11 Table. Reporting odds ratio and information components of HT for each drug as monotherapy. S12 Table. Reporting odds ratio and information components of ICH for each drug as monotherapy. (ZIP) [file pone.0329378.s001.zip › Supporting Information file/S12 Table.pdf]

S12 Table. Reporting odds ratio and information components of ICH for each drug as monotherapy.

Pharmacological classification	Drugs	N_{11}	N_{10}	N_{01}	N_{00}	ROR [95% CI]	IC [95% CI]	Signal
**Intravenous thrombolysis**								
tPA	Alteplase	232	671	9,201	795,840	29.91 [25.72 to 34.77]	4.33 [4.12 to 4.54]	Yes
**Antiplatelets**								
COX-1 inhibitor	Aspirin	311	19,814	9,122	776,697	1.34 [1.19 to 1.50]	0.40 [0.23 to 0.57]	Yes
$P2Y_{12}$ inhibitor	Clopidogrel sulfate	149	5,544	9,284	790,967	2.29 [1.94 to 2.70]	1.15 [0.91 to 1.39]	Yes
	Ticlopidine hydrochloride	27	1,529	9,406	794,982	1.49 [1.02 to 2.18]	0.54 [-0.01 to 1.09]	No
	Prasugrel hydrochloride	13	155	9,420	796,356	7.09 [4.02 to 12.49]	2.23 [1.43 to 3.04]	Yes
	Ticagrelor	0	4	9,433	796,507	0	-0.08 [-3.24 to 3.08]	No
PDE-3 inhibitor	Cilostazol	113	3,366	9,320	793,145	2.86 [2.37 to 3.45]	1.45 [1.17 to 1.73]	Yes
PDE inhibitor	Dipyridamole	6	1,128	9,427	795,383	0.45 [0.20 to 1.00]	-1.03 [-2.12 to 0.07]	No
TXA2 synthase inhibitor	Ozagrel sodium	12	252	9,421	796,259	4.02 [2.25 to 7.18]	1.66 [0.84 to 2.48]	Yes
PGE_1 analogue	Limaprost alfadex	29	3,049	9,404	793,462	0.80 [0.56 to 1.16]	-0.30 [-0.83 to 0.23]	No
PGI_2 analogue	Beraprost sodium	8	1,052	9,425	795,459	0.64 [0.32 to 1.29]	-0.58 [-1.54 to 0.39]	No
EPA	Ethyl icosapentate	17	2,374	9,416	794,137	0.60 [0.37 to 0.97]	-0.69 [-1.37 to -0.01]	No
EPA and DHA	Omega-3 fatty acid ethyl	8	594	9,425	795,917	1.14 [0.57 to 2.29]	0.16 [-0.81 to 1.13]	No
$5\text{-}HT_2$ receptor antagonist	Sarpogrelate hydrochloride	10	661	9,423	795,850	1.28 [0.68 to 2.39]	0.31 [-0.57 to 1.19]	No
Adenosine uptake inhibitor	Dilazep hydrochloride hydrate	4	367	9,429	796,144	0.92 [0.34 to 2.47]	-0.10 [-1.40 to 1.20]	No
PDGF antagonist	Trapidil	0	98	9,433	796,413	0	-1.11 [-4.01 to 1.79]	No
**Anticoagulants**								
DOACs	Apixaban	1,149	7,048	8,284	789,463	15.54 [14.54 to 16.60]	3.57 [3.47 to 3.66]	Yes
	Rivaroxaban	937	5,018	8,496	791,493	17.40 [16.17 to 18.71]	3.73 [3.62 to 3.83]	Yes
	Edoxaban tosilate hydrate	544	4,406	8,889	792,105	11.00 [10.04 to 12.06]	3.21 [3.08 to 3.34]	Yes
	Dabigatran etexilate methanesulfonate	318	2,765	9,115	793,746	10.02 [8.90 to 11.27]	3.10 [2.93 to 3.28]	Yes
Vitamin K antagonist	Warfarin potassium	420	10,173	9,013	786,338	3.60 [3.26 to 3.98]	1.75 [1.61 to 1.90]	Yes
Heparin group	Heparin sodium	138	5,189	9,295	791,322	2.26 [1.91 to 2.68]	1.13 [0.88 to 1.38]	Yes
	Heparin calcium	12	699	9,421	795,812	1.45 [0.82 to 2.57]	0.48 [-0.33 to 1.29]	No
Direct thrombin inhibitor	Argatroban	11	181	9,422	796,330	5.14 [2.79 to 9.44]	1.88 [1.02 to 2.74]	Yes

S12 Table (continued).

N_{11}, N_{10}, N_{01}, and N_{00} are shown in S3 Table, respectively. ICH, intracerebral hemorrhage; ROR, reporting odds ratio; IC, information component; CI, confidence interval; tPA, tissue-type plasminogen activator, COX-1, cyclooxygenase-1; PDE-3, phosphodiesterase-3; TXA2, thromboxane A2; PGE_1, prostaglandin E_1; EPA, ethyl icosapentate; DHA, docosahexaenoic acid; 5-HT_2, 5-hydroxytryptamine 2; PDGF, platelet-derived growth factor; DOACs, direct oral anticoagulants.
