# Supplementary material for: Analysis of hemorrhagic transformation and intracerebral hemorrhage under combination therapy with alteplase and antiplatelets or anticoagulants, using the Japanese Adverse Drug Event Report database
Source: PLoS One. 2025 Aug 18;20(8):e0329378. doi: 10.1371/journal.pone.0329378 (PMC12360569; doi:10.1371/journal.pone.0329378)
Supplement: S1 File — S1 Table. Definition of hemorrhagic transformation (HT). S2 Table. Definition of intracerebral hemorrhage (ICH). S3 Table. Two-by-two contingency table for adverse-event signal detection. S4 Table. Four-by-two contingency table for drug-drug interaction signal detection. S5 Table. Two-by-two contingency table for drug-drug interaction signal detection. S6 Table. Definition of hypertension. S7 Table. Definition of diabetes mellitus. S8 Table. Definition of heart failure. S9 Table. Definition of convulsions. S10 Table. Definition of chronic kidney disease. S11 Table. Reporting odds ratio and information components of HT for each drug as monotherapy. S12 Table. Reporting odds ratio and information components of ICH for each drug as monotherapy. (ZIP) [file pone.0329378.s001.zip › Supporting Information file/S4 Table.pdf]

S4 Table. Four-by-two contingency table for drug-drug interaction signal detection.

	Target AE	All other AEs	Total
Target drug D_1 and D_2	n_{111}	n_{110}	n_{11+}
Only drug D_1	n_{101}	n_{100}	n_{10+}
Only drug D_2	n_{011}	n_{010}	n_{01+}
Neither drug D_1 nor drug D_2	n_{001}	n_{000}	n_{00+}
Total	n_{++1}	n_{++0}	n_{+++}

AE, adverse event.
