# Supplementary material for: Analysis of hemorrhagic transformation and intracerebral hemorrhage under combination therapy with alteplase and antiplatelets or anticoagulants, using the Japanese Adverse Drug Event Report database
Source: PLoS One. 2025 Aug 18;20(8):e0329378. doi: 10.1371/journal.pone.0329378 (PMC12360569; doi:10.1371/journal.pone.0329378)
Supplement: S1 File — S1 Table. Definition of hemorrhagic transformation (HT). S2 Table. Definition of intracerebral hemorrhage (ICH). S3 Table. Two-by-two contingency table for adverse-event signal detection. S4 Table. Four-by-two contingency table for drug-drug interaction signal detection. S5 Table. Two-by-two contingency table for drug-drug interaction signal detection. S6 Table. Definition of hypertension. S7 Table. Definition of diabetes mellitus. S8 Table. Definition of heart failure. S9 Table. Definition of convulsions. S10 Table. Definition of chronic kidney disease. S11 Table. Reporting odds ratio and information components of HT for each drug as monotherapy. S12 Table. Reporting odds ratio and information components of ICH for each drug as monotherapy. (ZIP) [file pone.0329378.s001.zip › Supporting Information file/S6 Table.pdf]

S6 Table. Definition of hypertension.

SMQ code	SMQ name
20000147	Hypertension
PT code	PT name
10000358	Accelerated hypertension
10005732	Blood pressure ambulatory increased
10005739	Blood pressure diastolic increased
10051128	Blood pressure inadequately controlled
10005750	Blood pressure increased
10063926	Blood pressure management
10053355	Blood pressure orthostatic increased
10005760	Blood pressure systolic increased
10081751	Catecholamine crisis
10063067	Dialysis induced hypertension
10012758	Diastolic hypertension
10014129	Eclampsia
10057615	Endocrine hypertension
10015488	Essential hypertension
10070538	Gestational hypertension
10049058	HELLP syndrome
10020571	Hyperaldosteronism
10020772	Hypertension
10049781	Hypertension neonatal
10059238	Hypertensive angiopathy
10020801	Hypertensive cardiomegaly
10058222	Hypertensive cardiomyopathy
10077000	Hypertensive cerebrovascular disease
10020802	Hypertensive crisis
10058179	Hypertensive emergency
10020803	Hypertensive encephalopathy
10079496	Hypertensive end-organ damage
10020823	Hypertensive heart disease
10055171	Hypertensive nephropathy
10058181	Hypertensive urgency
10049079	Labile hypertension

S6 Table (continued).

10025600	Malignant hypertension
10025603	Malignant hypertensive heart disease
10026674	Malignant renal hypertension
10026924	Maternal hypertension affecting foetus
10026985	Mean arterial pressure increased
10052066	Metabolic syndrome
10067598	Neurogenic hypertension
10089290	Nocturnal hypertension
10065508	Orthostatic hypertension
10076704	Page kidney
10050631	Postoperative hypertension
10036485	Pre-eclampsia
10065918	Prehypertension
10062886	Procedural hypertension
10087816	Renal artery revascularisation
10038464	Renal hypertension
10074864	Renal sympathetic nerve ablation
10038562	Renovascular hypertension
10038926	Retinopathy hypertensive
10039808	Secondary aldosteronism
10039834	Secondary hypertension
10084825	Superimposed pre-eclampsia
10078932	Supine hypertension
10088977	Syndrome Z
10042957	Systolic hypertension
10048007	Withdrawal hypertension

SMQ, standardized Medical Dictionary for Regulatory Activities (MedDRA) queries; PT, preferred term.
