# Supplementary material for: Analysis of hemorrhagic transformation and intracerebral hemorrhage under combination therapy with alteplase and antiplatelets or anticoagulants, using the Japanese Adverse Drug Event Report database
Source: PLoS One. 2025 Aug 18;20(8):e0329378. doi: 10.1371/journal.pone.0329378 (PMC12360569; doi:10.1371/journal.pone.0329378)
Supplement: S1 File — S1 Table. Definition of hemorrhagic transformation (HT). S2 Table. Definition of intracerebral hemorrhage (ICH). S3 Table. Two-by-two contingency table for adverse-event signal detection. S4 Table. Four-by-two contingency table for drug-drug interaction signal detection. S5 Table. Two-by-two contingency table for drug-drug interaction signal detection. S6 Table. Definition of hypertension. S7 Table. Definition of diabetes mellitus. S8 Table. Definition of heart failure. S9 Table. Definition of convulsions. S10 Table. Definition of chronic kidney disease. S11 Table. Reporting odds ratio and information components of HT for each drug as monotherapy. S12 Table. Reporting odds ratio and information components of ICH for each drug as monotherapy. (ZIP) [file pone.0329378.s001.zip › Supporting Information file/S7 Table.pdf]

S7 Table. Definition of diabetes mellitus.

SMQ code	SMQ name
20000041	Hyperglycaemia/new onset diabetes mellitus
PT code	PT name
10087376	Acquired generalised lipodystrophy
10089543	Alpha hydroxybutyric acid increased
10065367	Blood 1,5-anhydroglucitol decreased
10005557	Blood glucose increased
10012596	Diabetes complicating pregnancy
10012601	Diabetes mellitus
10012607	Diabetes mellitus inadequate control
10012631	Diabetes with hyperosmolarity
10077357	Diabetic arteritis
10012650	Diabetic coma
10080788	Diabetic coronary microangiopathy
10071265	Diabetic hepatopathy
10012668	Diabetic hyperglycaemic coma
10012669	Diabetic hyperosmolar coma
10012671	Diabetic ketoacidosis
10012672	Diabetic ketoacidotic hyperglycaemic coma
10012673	Diabetic ketosis
10074309	Diabetic metabolic decompensation
10081558	Diabetic wound
10080061	Euglycaemic diabetic ketoacidosis
10017395	Fructosamine increased
10072628	Fulminant type 1 diabetes mellitus
10018209	Gestational diabetes
10018429	Glucose tolerance impaired
10018430	Glucose tolerance impaired in pregnancy
10018478	Glucose urine present
10082836	Glycated albumin increased
10087214	Glycated serum protein increased
10018473	Glycosuria
10018475	Glycosuria during pregnancy
10018481	Glycosylated haemoglobin abnormal

S7 Table (continued).

10018484	Glycosylated haemoglobin increased
10085610	Hepatogenous diabetes
10020635	Hyperglycaemia
10087319	Hyperglycaemic crisis
10063554	Hyperglycaemic hyperosmolar nonketotic syndrome
10071394	Hyperglycaemic seizure
10071286	Hyperglycaemic unconsciousness
10056997	Impaired fasting glucose
10022489	Insulin resistance
10022491	Insulin resistant diabetes
10053247	Insulin-requiring type 2 diabetes mellitus
10023379	Ketoacidosis
10023388	Ketonuria
10023391	Ketosis
10023392	Ketosis-prone diabetes mellitus
10066389	Latent autoimmune diabetes in adults
10086189	Maternally inherited diabetes and deafness
10075980	Monogenic diabetes
10028933	Neonatal diabetes mellitus
10086425	Neonatal hyperglycaemia
10082630	New onset diabetes after transplantation
10033660	Pancreatogenous diabetes
10087435	Pseudodiabetes
10081755	Steroid diabetes
10067584	Type 1 diabetes mellitus
10067585	Type 2 diabetes mellitus
10072659	Type 3 diabetes mellitus
10057597	Urine ketone body present

SMQ, standardized Medical Dictionary for Regulatory Activities (MedDRA) queries; PT, preferred term.
