# Supplementary material for: Analysis of hemorrhagic transformation and intracerebral hemorrhage under combination therapy with alteplase and antiplatelets or anticoagulants, using the Japanese Adverse Drug Event Report database
Source: PLoS One. 2025 Aug 18;20(8):e0329378. doi: 10.1371/journal.pone.0329378 (PMC12360569; doi:10.1371/journal.pone.0329378)
Supplement: S1 File — S1 Table. Definition of hemorrhagic transformation (HT). S2 Table. Definition of intracerebral hemorrhage (ICH). S3 Table. Two-by-two contingency table for adverse-event signal detection. S4 Table. Four-by-two contingency table for drug-drug interaction signal detection. S5 Table. Two-by-two contingency table for drug-drug interaction signal detection. S6 Table. Definition of hypertension. S7 Table. Definition of diabetes mellitus. S8 Table. Definition of heart failure. S9 Table. Definition of convulsions. S10 Table. Definition of chronic kidney disease. S11 Table. Reporting odds ratio and information components of HT for each drug as monotherapy. S12 Table. Reporting odds ratio and information components of ICH for each drug as monotherapy. (ZIP) [file pone.0329378.s001.zip › Supporting Information file/S8 Table.pdf]

**S8 Table. Definition of heart failure.**

| SMQ code | SMQ name |
|---|---|
| 20000004 | Cardiac failure |
| PT code | PT name |
| 10063081 | Acute left ventricular failure |
| 10001029 | Acute pulmonary oedema |
| 10063082 | Acute right ventricular failure |
| 10007522 | Cardiac asthma |
| 10007554 | Cardiac failure |
| 10007556 | Cardiac failure acute |
| 10007558 | Cardiac failure chronic |
| 10007559 | Cardiac failure congestive |
| 10007560 | Cardiac failure high output |
| 10007625 | Cardiogenic shock |
| 10082480 | Cardiohepatic syndrome |
| 10051093 | Cardiopulmonary failure |
| 10068230 | Cardiorenal syndrome |
| 10063083 | Chronic left ventricular failure |
| 10063084 | Chronic right ventricular failure |
| 10084058 | Congestive hepatopathy |
| 10010968 | Cor pulmonale |
| 10010969 | Cor pulmonale acute |
| 10010970 | Cor pulmonale chronic |
| 10050528 | Ejection fraction decreased |
| 10086366 | Heart failure with midrange ejection fraction |
| 10076396 | Heart failure with preserved ejection fraction |
| 10078289 | Heart failure with reduced ejection fraction |
| 10051448 | Hepatojugular reflux |
| 10024119 | Left ventricular failure |
| 10024899 | Low cardiac output syndrome |
| 10049780 | Neonatal cardiac failure |
| 10073708 | Obstructive shock |
| 10037423 | Pulmonary oedema |
| 10050459 | Pulmonary oedema neonatal |
| 10076203 | Radiation associated cardiac failure |

**S8 Table (continued).**

| | |
|---|---|
| 10075337 | Right ventricular ejection fraction decreased |
| 10039163 | Right ventricular failure |
| 10060953 | Ventricular failure |

SMQ, standardized Medical Dictionary for Regulatory Activities (MedDRA) queries; PT, preferred term.
